# Exploring the spatial heterogeneity in different doses of vaccination coverage in India

**DOI:** 10.1371/journal.pone.0207209

**Published:** 2018-11-28

**Authors:** Junaid Khan, Apurba Shil, Ravi Prakash

**Affiliations:** 1 International Institute for Population Sciences (IIPS), Mumbai, India; 2 Karnataka Health Promotion Trust (KHPT), Bangalore, India; Johns Hopkins Bloomberg School of Public Health, UNITED STATES

## Abstract

**Background:**

Despite the universalization of immunization against the six vaccine-preventable diseases (VPDs), the coverage of full immunization among the children under age five has remained a challenge globally. The 2015–16 National Family Health Survey (NFHS) indicated large disparity in the coverage of different vaccination doses (BCG, Polio, DPT and Measles) including full immunization across the districts of India. The spatial distribution of poor performing districts in terms of vaccination and the district level spatial, contextual determinants contributing to the low coverage have been poorly studied. Using the recent household survey (NFHS, 2015–16), this study examined the spatial heterogeneity and the factors associated with low vaccination coverage among the children aged 12–23 months across India.

**Data and methods:**

This study used the data from fourth round of National Family Health Survey conducted in 2015–16. District-level prevalence of each of the vaccination doses including full immunization, were analysed. Moran’s *I*, Univariate and Bivariate LISA, Ordinary least square (OLS) and spatial models were employed to achieve the overall aim of the study.

**Results:**

At the national level, the prevalence of full immunization was 62 percent. Specific vaccination coverage for BCG, three doses of polio, three doses of DPT and measles were 92, 73, 78 and 81 percent, respectively. The value of the bivariate Moran’s *I* statistics confirmed the spatial dependence between specific vaccination and the set of independent variables. District-level prevalence of the specific vaccine and full immunization showed significant spatial clustering across India. The adjusted coefficients from the spatial error model confirmed that district-level proportion of utilization of post-natal care, institutional births, neonatal tetanus protection of the last birth, women’s education and coverage of health insurance showed statistically significant association with every doses of vaccination coverage.

**Conclusion:**

The full and specific immunization coverage was considerably low in the geographical hotspots as compared to the national coverage. Maternal and child health care services utilization, financial assistance to the mothers through JSY scheme and mother’s education were found to determine full immunization as well as the specific vaccination coverage. Appropriate intervention should be designed to reduce the geographical disparity in the coverage of specific and full immunization across India and thus safeguard child health protecting the children from the vaccine preventable diseases across the geography.

## Background

Despite the fact that the universalization of immunization against six vaccine-preventable diseases (VPDs) (tuberculosis, diphtheria, pertussis, tetanus, polio, and measles) has been the most cost-effective intervention among the different public health interventions in developing countries over the last decade, the coverage of full immunization among children under the age of five years has remained a challenge globally [1,2]. While the burden of childhood morbidity and mortality due to these VPDs is quite high in developing countries, the childhood immunization program through public health programs largely aimed to reduce the morbidity and mortality among children specifically under the age of five years [3,4]. The guidelines given by the World Health Organization (WHO) on full immunization lays down the fact that children should receive all the three doses of Diphtheria, Pertussis, Tetanus (DPT) and polio vaccine and the vaccination against tuberculosis and measles. Despite these efforts, children from the developing countries like India, failed to complete the full course of immunization by the age of five due to various reasons including low parental awareness about immunization, parental literacy status, gender bias, caste differential and due to poverty [5]. The recent estimates on immunization coverage by WHO and UNICEF report that globally, nearly 1 in 10, about 12.9 million children did not receive any vaccine in the year 2016 and about 6.6 million children did not receive the first dose of DPT. Furthermore, despite the fact that immunization currently averts an estimated 2 to 3 million deaths every year, in the year 2016, around 19.5 million infants missed the routine immunization services and more than half (60 percent) of these children reside in 10 countries which include India, Pakistan, Indonesia, Angola, Brazil, the Democratic Republic of the Congo, Ethiopia, Iraq, Nigeria and South Africa [6,7].

Evidences suggest that the South Asian countries have made significant progress in bringing down child mortality rates from 129 per 1000 live births to 53 during the period from 1990 to 2015. Yet about two million children died before reaching the age of five in 2016 in this region and half of these deaths were due to VPDs [3,8]. India carries a significant burden of infant and under-five child mortality globally and the prevalence of vaccine-preventable diseases is one of the major underlying causes leading to high mortality. Recognizing the importance of full immunization, India launched a series of national programmes during1978 to 2013 to protect every child from the vaccine preventable diseases by ensuring full immunization. Despite having such an array of unique policies and programs, India could not achieve the goal 4 of the Millennium Development Goals (MDGs) and subsequently the goal 3 of Sustainable Development Goals (SDGs) which emphasize on good health and wellbeing. Combating the dropouts to different doses of full immunization is a major child health challenge common in majority of the South and South-East Asian countries and India is not an exception to this problem [8]. According to the recent National Family Health Survey (NFHS-4, 2015–16), 62 percent of children aged 12–23 months in India are fully vaccinated. Despite an increase in the immunization coverage from 19 percent to 43 percent over the last decade (NFHS-3, 2005–06), the percentage of children receiving full vaccination remained well under the target of 66 percent coverage set under the MDG-4, leaving behind 38 percent of the total children in India, who have either missed any of the doses of vaccination or never received any of the doses and are at higher risk of becoming infected with these fatal diseases.

Numerous studies have been conducted to examine the socio-demographic and economic determinants of child immunization in India and in other countries. The studies have explored a range of factors influencing full immunization coverage, however, they did not take into account the geographic variance in the prevalence of vaccination uptake [9–11]. Consequently, none of the previous studies identified the spatial heterogeneity and the associated distal determinants of full childhood vaccination at the district level. Furthermore, most of the existing studies looked at either full immunization as a whole or any of the specific vaccine components, but none of the existing studies in India have, so far, attempted to understand the distal determinants and the associated geographical context of every vaccination component under the full vaccination coverage, exclusively. To address this major research gap, this study is an attempt to assess the spatial heterogeneity and distal determinants of full immunization and its every vaccination dosage (Bacillus Calmette Guerin (BCG), D.P.T, polio and measles) using advanced geo-statistical methods. This study within the meso scale framework checks the possible effects of district-level utilization of maternal and child health (MCH) care services like antenatal care (ANC), institutional delivery and post-natal care (PNC) and mother’s educational status on every doses of vaccination and on full immunization. District has been taken as the unit of analysis and is used as the proxy for a geographical location to capture the geographical variability in the target and exposure variables, to critically examine the associations correcting the possible endogeneity due to spatial influence.

Assessing all the required vaccinations for full immunization and identifying factors that influence each vaccination component of full immunization at the district level will help researchers and policy makers understand the current situation, gaps and challenges. This would also help the National Health Policy for Childhood Immunization program in India to guide the design of interventions to reduce the gap in childhood vaccination. The geographic snapshots of child immunization can potentially help ensure that every child in India accesses full immunization leading to VPDs free and a healthy life. Additionally, insights from the report can be used by Government officials, researchers and policymakers to improve upon the current National Program on Immunization in India, strengthen existing policies and help design, pilot and implement cost-effective measures in smaller and remote areas to improve the coverage of routine immunization.

## Material & methods

### Data

The analysis of the study is based on the recently published data from the fourth round of National Family Health Survey (NFHS-4) conducted during 2015–16 which is available on the Demographic and Health Survey (DHS) website https://dhsprogram.com/data/dataset/India_Standard-DHS_2015.cfm?flag=1 and therefore, does not require any separate ethical approval. All four NFHS surveys have been conducted under the stewardship of the Ministry of Health and Family Welfare (MoHFW), Government of India (GOI). MoHFW designated the International Institute for Population Sciences (IIPS), Mumbai, as the nodal agency for all rounds of the survey. Like the earlier three rounds, this round also provides crucial insights on the different aspects of maternal, child, adolescent and adult health indicators. Additionally, it also provides clear estimates of malnutrition, anaemia, hypertension, HIV, and blood glucose levels through a series of biomarker tests and measurements.

A stratified two stage sampling design was adopted by considering the urban and rural areas as the natural strata. 2011 Census lists were used as the sampling frame for the selection of the sampling units. The Primary Sampling Units (PSUs) in the survey were villages in rural areas and Census Enumeration Blocks (CEBs) in urban areas. Within each rural stratum, PSUs were selected based on probability proportional to size (PPS) sampling from the sampling frame used. In urban areas, CEBs were also selected through PPS sampling. In every selected rural and urban PSUs, an estimated number of households were segmented. Then two of the segments were randomly chosen for the survey using systematic sampling with probability proportional to segment size. Therefore, theNFHS-4 cluster is either a PSU or a segment of a PSU. In the second stage, from the selected rural and urban clusters, households were randomly selected using systematic sampling. Four survey questionnaires (for households, women, men and biomarker questionnaire) were canvassed in 17 local languages using Computer Assisted Personal Interviewing (CAPI).

Total 601,509 households (HHs) were selected and from those households, a total of 699,686 eligible women (499,627 married) in the age group of 15–49 years and 112,122 eligible men (62,091 married) in the age group of 15–54 years were interviewed. All the digital maps were generated using the country representative district level shape file.

### Outcomes and predictors

The outcome variables used for this study are the district-wise proportions of: (1) children aged 12–23 months fully immunized (one dose of BCG, measles and three doses of polio and DPT according to the WHO schedule of immunizations), (2) children aged 12–23 months who received BCG, (3) children aged 12–23 months who received 3 doses of polio vaccine, (4) children aged 12–23 months who received 3 doses of DPT vaccine and (5) children aged 12–23 months who received measles vaccine.

After an exhaustive review of literature and following the frameworks of previous studies, we identified the set of independent variables for this specific study [12–16]. As the analysis was focused on the district-level disparities in immunization coverage using the available district-level aggregate data, we considered nine independent variables (predictors) of child immunization. These variables include the proportion of mothers who received postnatal care (PNC) from a doctor/nurse/LHV/ANM/midwife/other health personnel within two days of delivery, received full antenatal care (ANC), had institutional birth during the last birth, breastfed children aged 6–23 months receiving adequate diet, received Mother and Child Protection (MCP) card as a part of registering her pregnancy to a medical institution, had last birth protected against neonatal tetanus and received financial assistance under Janani Suraksha Yojana (JSY) for births delivered in an institution. JSY is a ‘Mother Security Scheme’ by the Government of India which aims to decrease maternal and neo-natal deaths among poor pregnant women, irrespective of age and number of children, by increasing institutional delivery through cash incentives. The cash incentives for mothers in the rural areas is INR 1400 (approx $20) in the low performing states (LPS) having low institutional delivery and it is INR 700 (approx$10) in high performing states having low institutional delivery rates, whereas the incentives are 1200 (approx $16) and 600 (approx $8) INR in the urban areas of low and high performing states respectively. Additionally, the analysis included two socio-economic characteristics at the household level, which are (1) proportion of women with 10 or more years of schooling and (2) proportion of households with any member covered by a health scheme or health insurance.

### India digital map

The district level shape file (digital map) of India was obtained from GitHub at https://github.com/datameet/maps/tree/master/Districts. The digital map has been used under the Creative Commons Attributions 2.5 India license. The shape file was created using the administrative atlas of Census 2011, India. And the map was projected in WGS 1984 UTM zone 43N.

### Analysis

A series of district-level quantile maps have been generated to understand the spatial pattern of child immunization coverage in India. To examine the spatial dependence and clustering of different doses of vaccination and full immunization coverage, Moran’s *I*, Univariate Local indicator of Spatial Association (LISA) cluster map and significance maps were produced. The Spatial weight matrix (*w*) of order 1 has been generated using the Queen’s contiguity method to quantify the spatial proximity between each possible pair of observational entities in the dataset [17,18]. Intuitively, Queen’s method defines neighbours as spatial units (districts) sharing a common boundary (a common edge or common vertex) of non-zero length. The Moran’s *I* measure of spatial autocorrelation indicates the degree to which data points are similar or dissimilar to their spatial neighbours [19]. So, to observe the spatial dependence of the components of immunization coverage across districts, univariate and bivariate local Moran’s *I* statistic values were computed. The formula to compute the Moran’s *I* statistic is as follows [20]:

$$\text{Univariate}\;\text{Moran}’\text{s}\;\text{I}=\frac{n}{S_{0}}\times \frac{\sum_{i}\sum_{j}W_{ij}\left(x_{i}-\bar{X}\right)\left(x_{j}-\bar{X}\right)}{\sum_{i}{\left(x_{i}-\bar{X}\right)}^{2}}$$

Where x is the variable of interest and $\bar{\text{x}}$ is the mean of x; n is the number of spatial units; *w*_*ij*_ is the standardized weight matrix between observation i and j with zeroes on the diagonal; and S_0_ is the aggregate of all spatial weights, i.e. ${\text{S}}_{0}=\Sigma_{i}\Sigma_{j}wij$.

Similarly, the bivariate Moran’s *I* statistic is expressed as follows:

$$\text{Bivariate}\;\text{Moran}’\text{s}\;\text{I}=\frac{n}{S_{0}}\times \frac{\sum_{i}\sum_{j}W_{ij}\left(x_{i}-\bar{X}\right)\left(y_{j}-\bar{Y}\right)}{\sum_{i}{\left(y_{i}-\bar{Y}\right)}^{2}}$$

Where x and y are the variables of interest; $\bar{\text{x}}$ is the mean of x; $\overset{-}{y}$ is the mean of y; n is number of spatial units; *w*_*ij*_ is the standardized weight matrix between observation i and j with zeroes on the diagonal; and S_0_ is the aggregate of all spatial weights, i.e. ${\text{S}}_{0}=\Sigma_{i}\Sigma_{j}wij$.

A positive spatial autocorrelation indicates that points with similar attribute values are closely distributed in space whereas negative spatial autocorrelation indicates that closely associated points are more dissimilar. Moran’s *I* usually takes values in between −1 to +1, where positive values suggest the spatial clustering of the similar values and negative values indicate the clustering of dissimilar values. A zero value indicates a random spatial pattern with no spatial autocorrelation. Univariate LISA measures the correlation of neighbourhood values around a specific spatial location [20]. It determines the extent of spatial randomness and clustering present in the data [21,22]. The measure (*I*_*i*_) is given by the following:

$$I_{i}=\frac{n.\;{(x}_{i}-\bar{\text{x}})}{\sum_{i}{{(x}_{i}-\bar{\text{x}})}^{2}}˟\sum_{j}w_{ij}{(x}_{j}-\bar{\text{x}})$$

Four types of spatial autocorrelation were generated based on the four quadrants of Moran’s scatter plots which are termed as follows:

- **Hot Spots**: Locations with high values, with similar neighbours (High-High).
- **Cold Spots**: Locations with low values, with similar neighbours (Low-Low).
- **Spatial Outliers**: Locations with high values, but with low-value neighbours (High-Low) and locations with low values, but with higher values of neighbours (Low-High).

In addition to univariate LISA, bivariate LISA measures were estimated to analyse the association of certain characteristics of regions with different outcomes of child immunization. The bivariate LISA presented as below:

$$I_{i}=\frac{n.\;{(x}_{i}-\bar{\text{x}})}{\sum_{i}{{(y}_{i}-\overset{-}{y})}^{2}}˟\sum_{j}w_{ij}{(y}_{j}-\overset{-}{y})$$

Using the LISA functions in Geo-Da environment, cluster and significant maps were generated. The cluster map is a special choropleth map. The map identifies those locations having a significant local Moran’s *I* statistic classified by the type of spatial autocorrelation where red color represents the Hotspots, deep blue represents the Cold Spots, and light blue and light red color represents the Spatial Outliers. To examine the potential correlates of different vaccination doses along with full immunization, we performed a set of standard statistical regressions. First, we employed the ordinary least square (OLS) model to check the primary association between the independent variables and the outcomes of interest and further estimated the spatial autocorrelation in the residuals from the OLS model to examine the endogeneity due to spatial dependence. As we found the corresponding Moran’s I value statistically significant for each of the outcomes, we then further estimated the spatial autoregressive models- spatial lag and spatial error model to get the unbiased estimates of the associations between the outcomes and the predictors correcting the spatial endogeneity present in the data. In the case of a typical spatial lag model, it is assumed that the observations are spatially dependent and lagged to each other in the neighbourhood areas whereas spatial error model assumes that the residuals are correlated in the neighbourhood of the spatial units. Spatial error model unlike the spatial lag model takes into account the effect of the omitted variables (spatially correlated) which are not present in the model but may affect the estimation. Diagnostics tests for both the models were carried out and the value of Lagrange Multiplier of each of the models was found statistically significant. Next we compared the AIC values to identify the final model and we found error model to be the best fit for this specific study. The following are the functional forms of the statistical regressions employed in this specific study. The basic equation of linear regression (OLS) can be defined as:

Y=α+βX+ε

Where Y is the dependent variable, x is the vector of explanatory variables, α is the model intercept and β is the corresponding coefficient vector. It is assumed that disturbance term (ε) is identically and independently distributed (i.i.d). If spatial dependence is found to be significant, the OLS would provide biased and inconsistent estimates of the model parameters due to simultaneity bias. However, to control the spatial effects, spatial autoregressive (SAR) models are fitted as follows:

Spatial Lag Model (SLM), if the dependent variable (Y), is correlated with the weighted average of its value in its neighbourhood and other locations where ρ is the spatial lag parameter, this relationship can be expressed as follows:

$$Y=\rho w_{y}+\beta x+\varepsilon$$

Spatial Error Model (SEM), if spatial dependence enters through residuals, then the following model appropriately controls the spatial effect:

$$Y=\beta x+\varepsilon ,\;\text{where}\;\varepsilon =\lambda w_{\varepsilon }+\zeta$$

Here, λ is the spatial auto-regressive parameter and the error ζ is independently and identically distributed. Both spatial error and spatial lag models are estimated by maximizing the corresponding likelihood functions [23]. Stata version 12.0 (StataCorp^™^, Texas); Arc-GIS version 10.1, Esri, California and Geo-Da version 1.12.1.129, Teknowledgist, New York were used for the analysis.

## Results

Table 1 gives the summary measure of the dependent variables and the independent variables used in this study. Results show that 62 percent of the total children under the age of five were fully immunized. Among the different doses of immunization, coverage of BCG was the highest (92 percent) while the coverage of polio vaccine was the lowest (73 percent). The total coverage of DPT and measles across India were 78 and 81 percent respectively, depicting better coverage than polio.

**Table 1:** Coverage of specific immunization doses, full immunization and levels of selected independent variables, NFHS-4, 2015–16, India.

Dependent Variables	Percentage
Children aged 12–23 months who have received BCG vaccine	91.9
Children aged 12–23 months who have received polio vaccine	72.8
Children aged 12–23 months who have received DPT vaccine	78.4
Children aged 12–23 months who have received measles vaccine	81.1
Children aged 12–23 months who are fully immunized	62.0
**Independent variables (Predictors)**	
Mothers who received postnatal care (PNC) from a doctor/nurse/LHV/ANM/midwife/other health personnel within 2 days of delivery	62.4
Mothers who had full Antenatal Care (ANC)	21.0
Institutional births	78.9
Breastfeeding children age 6–23 months receiving an adequate diet	8.7
Registered pregnancies for which the mother received Mother and Child Protection (MCP) card	89.3
Mothers whose last birth was protected against neonatal tetanus	89.0
Mothers who received financial assistance under Janani Suraksha Yojana (JSY) for births delivered in an Institution	36.4
Women with 10 or more years of schooling	35.7
Household with any usual member covered by a health scheme or health insurance	28.7
Total no of children aged 12–23 months	48,928

**Fig 1:**
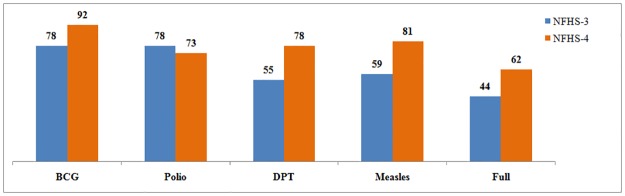
Child immunization coverage in India, National Family Health Survey (NFHS), 2005–06 & 2015–16.

Fig 1 gives the comparison in the coverage of the different vaccine doses for the two different survey periods, NFHS, 2005–06 & 2015–16. It is evident that coverage of full immunization has increased from 44 to 62 percent during the period of 2005 to 2016. Though the different doses of full immunization has seen an increase in the coverage, the coverage of polio vaccine has seen a five percentage point drop (78 percent in 2005–06 to 73 percent in 2015–16) in the last decade. The comparative visualization of the data also indicates that DPT coverage has seen maximum increase followed by measles and BCG in the last decade. Descriptive statistics of the independent variables used in this study shows that 62 percent of the mothers utilized post-natal care, 21 percent had full antenatal care, the coverage of institutional birth for the last delivery was 79 percent, of the registered pregnancies, 89 percent of the mothers received mother and child protection card, and similar proportion of the mothers’ last birth was protected against neonatal tetanus and 36 percent of the mothers received financial assistance under Janani Suraksha Yojana (JSY). Around 36 percent of the women completed 10 years of schooling and around 29 percent of the households with any usual member are found to be covered by a health scheme or health insurance.

### Spatial pattern and clustering of different vaccination doses and full immunization

**Fig 2:**
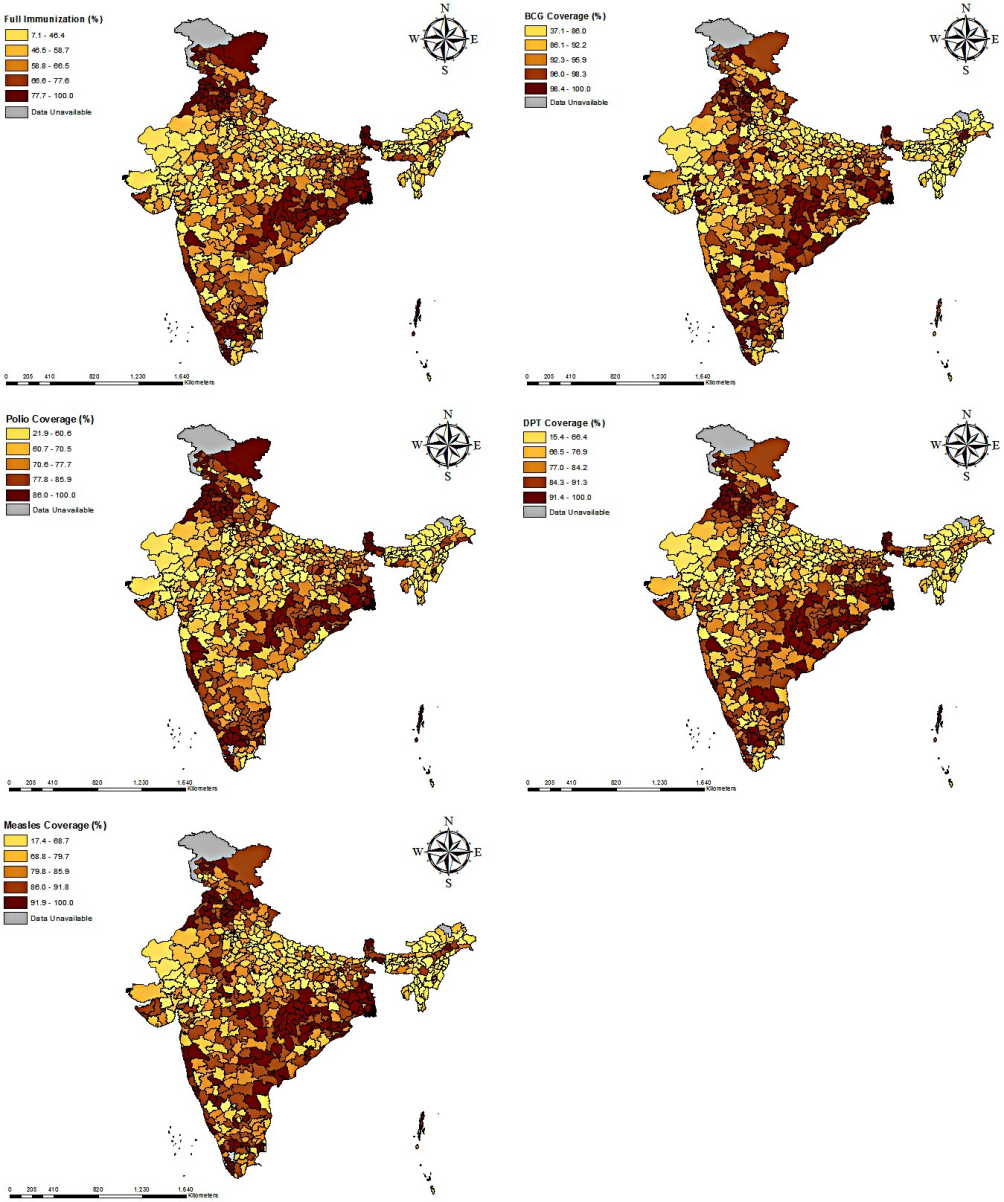
Rates of different doses of vaccination coverage among the children aged 12–23 months across the districts of India, 2015–16. (A) Full Immunization. (B) BCG Vaccination. (C) Polio Vaccination. (D) DPT Vaccination. (E) Measles Vaccination.

Fig 2A–2E show the coverage and spatial distribution of full immunization, BCG, DPT, Polio and Measles across the districts of India. The colour pattern shows the spatial differences in the immunization coverage. While the deeper color indicates a higher proportion of immunization coverage, the light colour indicates the lower coverage. From the spatial maps it is evident that there is a geographical disparity in the coverage of full immunization and its different doses across the districts. Overall, the observed spatial pattern of BCG, Polio, DPT and Measles vaccination coverage were consistent in the most parts of India. For example, the western part of India (Rajasthan and some of the districts of Gujarat and Maharashtra), north-eastern part (Arunachal Pradesh, Nagaland, Manipur, Mizoram, Assam, Tripura and Meghalaya) and some of the districts from the states of central India like Madhya Pradesh, Uttar Pradesh, and Bihar had a relatively lower coverage of every vaccination dose, including full immunization, as compared to the national average presented in Fig 1. On the other hand, the states located in the northern part of the country (Himachal Pradesh, Jammu & Kashmir, and some parts of Punjab and Haryana), eastern India (West Bengal, Sikkim, Orissa, and some parts of Chhattisgarh) and southern parts (Kerala, Tamil Nadu, Karnataka and some parts of Andhra Pradesh and Telangana of India showed relatively higher coverage of vaccination doses than the rest of India. Table 2 lists out the specific districts across the states of India identifying the top ten highest and lowest districts in terms of the coverage of every vaccination doses.

**Table 2:** Coverage of vaccination in percentages, ten lowest and highest districts across India, NFHS-4, 2015–16.

**Lowest**	**BCG coverage(%)**	**Highest**	**BCG coverage (%)**
East Kameng	37.2	Mahbubnagar	100
Bahraich	44.0	Hazaribagh	100
Mon	48.7	Khammam	100
Kurung Kumey	49.2	Bangalore Rural	100
Wokha	50.1	Srikakulam	100
Balrampur	54.2	Davanagere	100
Longleng	55.4	Thiruvananthapuram	100
Shrawasti	58.1	The Nilgiris	100
Upper Subansiri	60.4	Kozhikode	100
Nicobars	61.1	Gurdaspur	100
**Lowest**	**DPT coverage (%)**	**Highest**	**DPT coverage (%)**
Bahraich	15.4	Kottayam	100
East Kameng	17.4	Hugli	100
Balrampur	18.2	Dhenkanal	100
Mewat	23.8	Kapurthala	100
Shrawasti	27.4	Nadia	100
Longleng	28.2	Bankura	100
KurungKumey	28.6	Moga	100
Mon	30.6	Panchkula	100
Dhubri	30.7	Ambala	100
Upper Subansiri	31.1	Faridkot	100
**Lowest**	**Polio coverage (%)**	**Highest**	**Polio coverage (%)**
Balrampur	21.9	Ambala	100
Bahraich	25.7	Faridkot	100
East Kameng	27.6	Moga	100
Mon	29.0	Muktsar	100
Jhabua	30.7	Kapurthala	100
KurungKumey	31.0	Kottayam	100
Dhubri	33.7	Tarn Taran	100
Zunheboto	36.2	Barnala	98.7
Mewat	36.7	Bankura	98.0
KarbiAnglong	38.4	Tiruppur	97.9
**Lowest**	**Measles coverage (%)**	**Highest**	**Measles coverage (%)**
East Kameng	17.4	Panchkula	100
Bahraich	27.0	Krishna	100
KurungKumey	28.2	Kinnaur	100
Mon	31.1	Kapurthala	100
Balrampur	31.4	North District	100
Mewat	32.3	South District	100
Shrawasti	37.3	Kurukshetra	100
Longleng	37.9	South Goa	98.9
Dhubri	38.5	Birbhum	98.5
Upper Subansiri	41.9	Uttar BastarKanker	98.5
**Lowest**	**Full immunization (%)**	**Highest**	**Full immunization (%)**
Balrampur	7.1	Kapurthala	100.0
Bahraich	9.4	Faridkot	97.8
Longleng	10.8	Ambala	97.4
East Kameng	11.9	Panchkula	96.9
Mewat	13.1	Muktsar	96.9
Shrawasti	17.3	Tarn Taran	96.5
KurungKumey	17.4	Bankura	96.2
Mon	19.9	Patiala	95.3
Dhubri	20.1	Kottayam	95.2
Upper Subansiri	21.9	South Twenty Four Parganas	94.8

**Fig 3:**
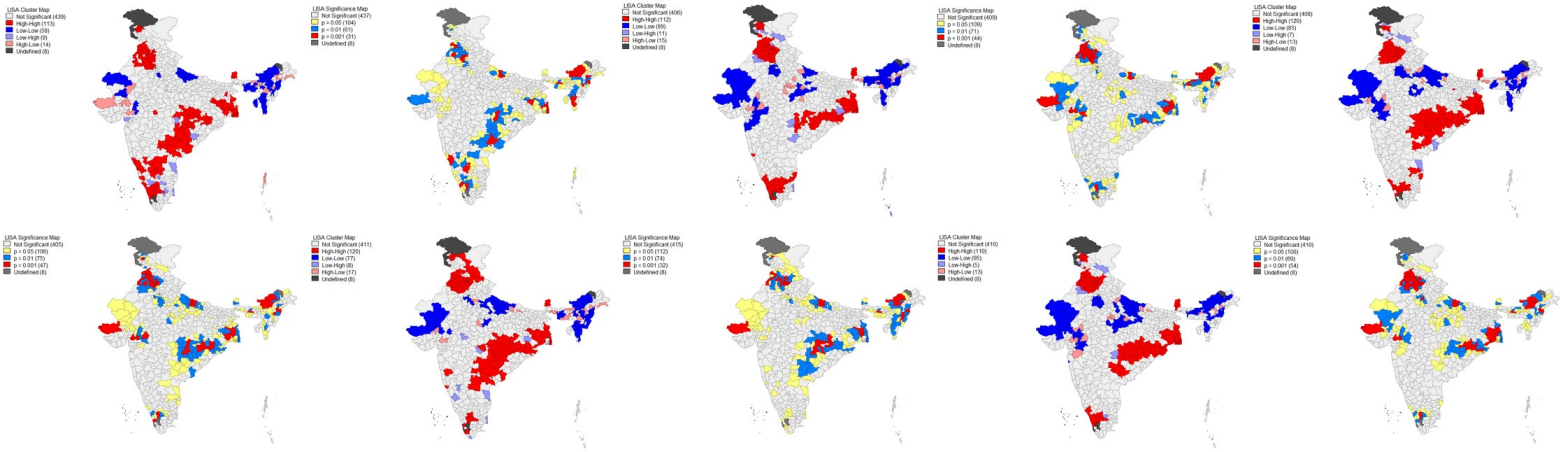
Univariate LISA cluster and significance maps showing the spatial clustering and outliers of different doses of vaccination among the children aged 12–23 months across the districts of India, 2015–16. (A) Cluster map of BCG coverage (Local Moran’s I = 0.48, p-value<0.001. (B) Significance map of BCG coverage. (C) Cluster map of Polio coverage (Local Moran’s I = 0.54, p-value<0.001). (D) Significance map of Polio coverage. (E) Cluster map of DPT coverage (Local Moran’s I = 0.557, p-value<0.001). (F) Significance map of DPT coverage. (G) Cluster map of Measles coverage (Local Moran’s I = 0.518, p-value<0.001). (H) Significance map of Measles coverage. (I) Cluster map of full immunization coverage (Local Moran’s I = 0.556, p-value<0.001). (J) Significance map of full immunization coverage.

Estimated Moran’s *I* statistic values indicate a highly significant spatial dependence in the coverage of different vaccination doses including full immunization across the districts of India. LISA cluster and significance map in Fig 3A, 3C, 3E, 3G & 3I showed a total of 58 (BCG), 89 (Polio), 85 (DPT), 77 (Measles) and 95 (full immunization) cold spots in Rajasthan, Uttar Pradesh and in North-Eastern states (Arunachal Pradesh, Assam, Nagaland, Manipur, Mizoram and Meghalaya) while some parts of Madhya Pradesh and Gujarat are showing substantially low levels of immunization coverage. Similarly, a total of 113 (BCG), 112 (Polio), 120 (DPT), 120 (Measles) and 110 (full immunization) hot spots were found in the states of Punjab, Himachal Pradesh, Haryana, West Bengal, some parts of Orissa, Chhattisgarh, Andhra Pradesh, Telangana, Kerala, Karnataka, Tamil Nadu, Sikkim and Jammu & Kashmir.

Bivariate LISA estimates of spatial autocorrelation presented in Table 3 demonstrate compelling evidence of spatial autocorrelation between the different components of childhood immunization (BCG, Polio, DPT and Measles) with most of the exposure variables considered in this study. Of the different exposure variables, post-natal care (PNC) utilization, utilization of full ANC care, institutional delivery and neonatal tetanus protection consistently showed a positive and statistically significant spatial autocorrelation with each of the vaccination doses along with full immunization. In case of BCG, mothers who received financial assistance showed an insignificant spatial autocorrelation, breastfed children who received adequate diet showed a very low (insignificant) and negative autocorrelation and the variables like-registered pregnancies for which the mother received financial assistance and health insurance showed low spatial autocorrelation. In case of polio, mothers who received financial assistance showed negative but significant autocorrelation. Likewise, the pattern of spatial autocorrelation (and the statistical significance) between all the vaccination doses and exposure variables are shown in Table 3.

**Table 3:** Moran’s *I* statistics showing bivariate spatial association between immunization and their predictors, 2015–16, India, (NFHS-4).

Predictors	BCG	Polio	DPT	Measles	Full Immunization
PNC (%)	0.37 (0.001)	0.40 (0.001)	0.40 (0.001)	0.42 (0.001)	0.42 (0.001)
Full ANC (%)	0.25 (0.001)	0.33 (0.001)	0.34 (0.001)	0.32 (0.001)	0.34 (0.001)
Institutional Births (%)	0.35 (0.001)	0.31 (0.001)	0.34 (0.001)	0.38 (0.001)	0.32 (0.001)
Breastfed child receive adequate diet (%)	-0.01 (0.417)	0.14 (0.001)	0.09 (0.001)	0.04 (0.046)	0.14 (0.001)
MCP Card (%)	0.17 (0.001)	0.18 (0.001)	0.24 (0.001)	0.28 (0.001)	0.22 (0.001)
Neonatal Tetanus (%)	0.34 (0.001)	0.26 (0.001)	0.35 (0.001)	0.33 (0.001)	0.30 (0.001)
Mothers who received financial assistance (%)	-0.03 (0.154)	-0.09 (0.001)	-0.04 (0.027)	-0.04 (0.003)	-0.05 (0.004)
Women with 10+ years of schooling (%)	0.20 (0.001)	0.32 (0.001)	0.23 (0.001)	0.22 (0.001)	0.26 (0.001)
Health Insurance (%)	0.09 (0.001)	0.15 (0.001)	0.20 (0.001)	0.18 (0.001)	0.18 (0.001)

Each cell shows the corresponding value of bivariate Local Moran’s I statistic and the values within parenthesis are the p-values.

### Estimated results from the OLS and spatial regression (SAR) models

Results from OLS and SAR models describing the effect of the predictors on the outcomes, after adjusting for the spatial effect are presented in Table 4(a)–4(c). Based on the model diagnostics and comparing the Akaike Information criterion (AIC) values, the Spatial Error Model (SEM) was found to be the best-fit model for all the outcome variables used in this study.

**Table 4:** OLS, spatial lag & spatial error model estimation of BCG and polio vaccination, India, 2015–16.

Predictors	BCG Vaccine*			Polio Vaccine*		
	OLS	SLM	SEM	OLS	SLM	SEM
PNC (%)	0.09 (0.003)	0.06 (0.016)	0.08 (0.008)	0.31 (0.000)	0.20 (0.000)	0.20 (0.000)
Full ANC (%)	-0.02 (0.464)	-0.02 (0.536)	0.02 (0.557)	0.00 (0.963)	0.00 (0.996)	0.06 (0.154)
Institutional Births (%)	0.08 (0.004)	0.06 (0.030)	0.08 (0.018)	-0.08 (0.072)	-0.04 (0.352)	0.06 (0.239)
Breastfed child received adequate diet (%)	0.07 (0.067)	0.07 (0.052)	0.01 (0.880)	0.33 (0.000)	0.25 (0.000)	0.20 (0.002)
MCP Card (%)	0.03 (0.311)	0.03 (0.210)	0.09 (0.005)	0.09 (0.054)	0.07 (0.092)	0.15 (0.003)
Neonatal Tetanus (%)	0.43 (0.000)	0.34 (0.000)	0.39 (0.000)	0.41 (0.000)	0.29 (0.000)	0.27 (0.000)
Mothers received financial assistance (%)	0.03 (0.030)	0.03 (0.044)	0.03 (0.074)	0.03 (0.164)	0.02 (0.283)	0.04 (0.212)
Women schooling 10+ years (%)	0.11 (0.000)	0.09 (0.001)	0.09 (0.007)	0.23 (0.000)	0.12 (0.001)	0.11 (0.031)
Health Insurance (%)	0.04 (0.004)	0.03 (0.023)	0.04 (0.042)	0.04 (0.065)	0.02 (0.400)	0.02 (0.368)
**N**	633	633	633	633	633	633
**ρ**		0.37(0.00)			0.48(0.00)	
**λ**			0.52 (0.00)			0.58 (0.00)
**AIC**	4192.5	4108.2	4073.7	4756.9	4618.1	4616.9
**Adjusted R**^**2**^	0.521	0.594	0.627	0.468	0.601	0.612
OLS-Ordinary Least Square Model, SLM-Spatial Lag Model, SEM-Spatial Error Model						

*Each cell gives the estimated coefficients from the regression models and the values within parenthesis are the p-values.

### BCG vaccination

Table 4 gives the estimated results of OLS, SLM and SEM models for BCG vaccination across the districts. Though the explained variabilities of the models were not directly comparable due to the nature of the estimation procedure, the introduction of spatial models could better predict the variability of BCG vaccination coverage across districts as evident through the improved pseudo R-square and AIC values of the models. Postnatal care utilization, institutional delivery, mother’s last birth being neonatal tetanus protected, financial assistance, women’s education and health insurance scheme were consistently associated with BCG vaccination coverage throughout the models. Among the set of predictors, neonatal tetanus protection showed the strongest association with BCG vaccine coverage and a 10 percentage point increase in the proportion of neonatal tetanus protection for the last birth was significantly associated with 4.3 percent increase in the BCG vaccination coverage (SEM model). Similarly, with an estimated 10 percentage point increase in the proportion of 10+ years educated women was statistically associated with 1 percentage point increase in the BCG vaccination coverage. The value of the lag coefficient (0.18, p-value <0.001) from the spatial lag model suggested that a change in the BCG vaccine coverage in a particular district may statistically lag the rate of BCG coverage by 37 percent in the neighbouring districts. The final SEM model suggested that except the proportion of breastfed children who received adequate diet and full ANC, all the remaining predictors had positive impact on BCG vaccination coverage (Table 4). Comparing all the models for BCG, we found the SEM model to be the best fit and the associated error lag value of the model 0.53 (p-value <0.001) indicated the spatial influence on BCG coverage through the omitted variables not present in the SEM model.

### Polio vaccination

The estimated coefficients from OLS, SLM and SEM model gives the level of associations between the variables with Polio vaccination coverage across the districts (Table 4). Postnatal care, children who are breastfed and received adequate diet, mothers whose last birth is tetanus protected and women with 10 or more years of schooling are the factors which showed a consistent association throughout the models, significantly determining the coverage of polio vaccine across the districts of India. From the SEM model estimates it is observed that a 10 percent increase in postnatal care utilization among mothers is associated with 2 percent increase in the coverage of polio vaccine. Child’s breastfeeding practice across the districts showed a close association with the coverage of polio vaccine and it is found that districts where the proportion of children who are breastfed and received adequate diet showed better coverage of polio vaccination. Similarly, a 10 percent increase in the proportion of mothers whose last birth is neonatal tetanus protected is associated with 3 percent increase in the polio vaccine coverage (SEM estimates, Table 4). District-wise proportion of registered pregnancies for which the mother received MCP card also showed a significant association with polio vaccine coverage. Throughout the models, women’s educational status has been found to be one of the significant predictors of polio vaccination coverage across India. According to the SEM model, 10 percent increase in the proportion of women with 10 or more years schooling is associated with 1 percent increase in the polio vaccine coverage and the lag coefficient from the SLM model for polio vaccine coverage has been found to be 30 percent. Finally, correcting the spatial endogeneity through the spatial models, postnatal care utilization among mothers, breastfeeding pattern, registered pregnancies for which the mother received MCP card, neonatal tetanus protection and women’s education emerged to be the strongest predictors of polio vaccination coverage across India’s districts. Again the SEM model found to be the best fit with the lowest AIC value of 4616.9 and the corresponding error lag value (**λ)** was 0.58 (p-value<0.001).

### DPT vaccination

The estimated output from the OLS and spatial models for DPT vaccination coverage and its determinants are shown in Table 5. The preliminary OLS regression analysis shows that neonatal tetanus protection of the last birth, breastfeeding pattern across the districts, registered pregnancies for which the mother received MCP card, women’s educational status in the districts, postnatal care utilization among mothers and health insurance scheme show statistically significant association with the DPT vaccination coverage across India. Introduction of spatial models in the data helped us to consider the spatial dependence and to correct the estimates from the OLS model. Both spatial lag and error models gave improved estimates and comparing the AIC value for both the models we have found that the SEM model gives the better fit in this case. The value of adjusted R-square also confirmed that the spatial error model gave the best fit and explained the maximum variability compared to the other models. Both the spatial models suggested that neonatal tetanus protection against the last birth, breastfeeding pattern, MCP card, women’s education and health insurance schemes are the strongest predictors of DPT vaccine coverage across the geography of India. The lag coefficient (**ρ**) for DPT vaccine has been found to be 44percent (SLM model, Table 5) whereas the corresponding error lag value (λ) of the SEM model was 62 percent (0.62, p-value<0.001).

**Table 5:** OLS, spatial lag & spatial error model estimation of DPT and measles vaccination, India, 2015–16.

Predictors	DPT Vaccine*			Measles Vaccine*		
	OLS	SLM	SEM	OLS	SLM	SEM
PNC (%)	0.16 (0.000)	0.10 (0.004)	0.10 (0.008)	0.14 (0.000)	0.09 (0.010)	0.072 (0.054)
Full ANC (%)	0.07 (0.090)	0.05 (0.190)	0.10 (0.011)	0.03 (0.456)	0.02 (0.464)	0.074 (0.048)
Institutional Births (%)	-0.02 (0.674)	0.00 (0.964)	0.08 (0.065)	0.08 (0.040)	0.06 (0.055)	0.139 (0.001)
Breastfed child received adequate diet (%)	0.31 (0.000)	0.24 (0.000)	0.16 (0.004)	0.14 (0.007)	0.13 (0.009)	0.056 (0.304)
MCP Card (%)	0.19 (0.000)	0.15 (0.000)	0.26 (0.000)	0.17 (0.000)	0.12 (0.001)	0.168 (0.000)
Neonatal Tetanus (%)	0.68 (0.000)	0.50 (0.000)	0.46 (0.000)	0.56 (0.000)	0.45 (0.000)	0.493 (0.000)
Mothers received financial assistance (%)	0.06 (0.005)	0.05 (0.004)	0.09 (0.000)	0.05 (0.020)	0.04 (0.020)	0.056 (0.017)
Women schooling 10+ years (%)	0.17 (0.000)	0.14 (0.000)	0.19 (0.000)	0.13 (0.000)	0.10 (0.002)	0.098 (0.022)
Health Insurance (%)	0.09 (0.000)	0.05 (0.003)	0.06 (0.010)	0.08 (0.000)	0.06 (0.001)	0.082 (0.000)
**N**	633	633	633	633	633	633
**ρ**		0.44(0.00)			0.37 (0.00)	
**λ**			0.62 (0.00)			0.50 (0.00)
**AIC**	4635.5	4500.3	4474.1	4530.3	4442.9	4423.9
**Adjusted R**^**2**^	0.602	0.693	0.718	0.579	0.650	0.669
OLS-Ordinary Least Square Model, SLM-Spatial Lag Model, SEM-Spatial Error Model						

*Each cell gives the estimated coefficients from the regression models and the values within parenthesis are the p-values.

### Measles vaccination

Table 5 also shows the estimated coefficients from the OLS and spatial models for measles vaccination coverage. Estimates from the OLS model showed that except ANC care utilization, every variable under this study framework showed a positive association with the coverage of measles vaccine in the districts. From the OLS estimation, it was found that neonatal tetanus protection, breastfeeding pattern, MCP card, postnatal care utilization and women’s education showed a dominant association to determine the pattern of measles vaccination rate across the districts. The pattern of association remained the same after introducing the spatial models. The lag coefficient for measles vaccine in the SLM model has been found to be 37percent across the districts. The final SEM model suggested that a 10 percent increase in the proportion of neonatal tetanus protection across the districts is associated with 5 percent increase in the measles vaccination. However, except the proportion of breastfed children receiving adequate diet all the remaining variables showed a significant impact on measles vaccination coverage. And among the predictors, the proportion of mothers whose last birth was protected against neonatal tetanus, the proportion of registered pregnancies for which the mothers received MCP card, institutional births and women’s education with 10+ years of schooling showed higher association with the Measles vaccination coverage. The corresponding AIC values for the SLM and SEM models were found to be 4442.9 and 4423.9 respectively. And the error lag value of the SEM model was 50 percent (0.50, p-value<0.001).

### Full immunization

Finally, we estimated the district-level coverage of full immunization and assessed the determinants of full immunization across districts of India. The estimated coefficients from the OLS and spatial models are shown in Table 6. According to the OLS estimation, postnatal care, breastfeeding pattern, having MCP card, neonatal tetanus protection, and women’s education were found to be the dominant predictors of full immunization and were substantially associated with the increased coverage of full immunization. The estimated coefficients were 0.34 (p-value<0.001) for PNC, 0.45 (p-value<0.001) for breastfeeding pattern, 0.19 (p-value<0.001) for having MCP card, 0.62 (p-value<0.001) for neonatal tetanus protection and 0.19 (p-value<0.001) for women’s education and the coefficients were highly statistically significant. After spatial adjustments through spatial lag and error model, it is found that the pattern of association remained the same between full immunization and its predictors. Though the predictors remained statistically significant, after the spatial endogeneity error correction, we obtained refined estimates of the coefficients for the predictors. Comparing the AIC values for both the spatial models we have found that the error model gave the best fit in case of full immunization also. The estimate of spatial lag coefficient from the SLM model was found to be 0.45for full immunization which is more than that of the independent vaccination doses. On the other hand, the error lag value of the SEM model was found to be 0.59 (p-value<0.001). It is also observed that, there has been no significant change found while determining the predictors of full immunization from that of the separate vaccination doses. In this context, the final SEM model indicated that except the proportion of institutional births and health insurance variables, all the remaining predictors had a significant effecton achieving full immunization across the districts. And the SEM model showed the lowest AIC value compared to every other model.

**Table 6:** OLS, spatial lag & spatial error model estimation of full immunization, India, 2015–16.

Predictors	Full Immunization*		
	OLS	SLM	SEM
PNC (%)	0.34 (0.000)	0.22 (0.000)	0.19 (0.000)
Full ANC (%)	0.09 (0.007)	0.08 (0.078)	0.17 (0.001)
Institutional Births (%)	-0.12 (0.018)	-0.06 (0.174)	0.06 (0.299)
Breastfed child received adequate diet (%)	0.45 (0.000)	0.34 (0.000)	0.28 (0.000)
MCP Card (%)	0.19 (0.000)	0.14 (0.003)	0.25 (0.000)
Neonatal Tetanus (%)	0.62 (0.000)	0.46 (0.000)	0.44 (0.000)
Mothers received financial assistance (%)	0.07 (0.010)	0.05 (0.022)	0.08 (0.012)
Women schooling 10+ years (%)	0.19 (0.000)	0.12 (0.006)	0.13 (0.031)
Health Insurance (%)	0.07 (0.009)	0.03 (0.185)	0.04 (0.162)
**N**	633	633	633
**ρ**		0.45 (0.00)	
**λ**			0.59 (0.00)
**AIC**	4903.2	4765.9	4756.1
**Adjusted R**^**2**^	0.55	0.660	0.677
OLS-Ordinary Least Square Model, SLM-Spatial Lag Model, SEM-Spatial Error Model			

*Each cell gives the estimated coefficients from the regression models and the values within parenthesis are the p-values.

## Discussion

Using the district-level data from the most recent NFHS in India, this study attempted to assess the coverage and district level distal determinants of full immunization and its different doses (BCG, Polio, DPT and Measles) among the children aged 12–23 months across the districts of India. We found that only 62 percent of the total children were fully immunized. Not only that, among the different doses of routine immunization, BCG showed the highest level of coverage (92 percent) followed by Measles (81 percent), DPT (78 percent). Despite the fact that the Indian immunization programme had a lot of focus on Polio eradication, just 73% children aged 12–23 months were vaccinated for polio as per the recent estimate. It was also observed that the coverage rate of polio vaccine has seen a decline from 78 percent to 73 percent from 2005–06 to 2015–16. To speed up the coverage of essential health services in India, several efforts have been initiated including the Universal Immunization Program (UIP, 1985), the Childhood Survival and Safe Motherhood Program (CSSM, 1992), the National Health Policy (NHP, 2002), and the National Rural Health Mission (NRHM, 2005). The UIP in India is one of the largest public health intervention in the World in terms of beneficiaries, number of immunization sessions, quantities of vaccines used, and the geography it covers. Though UIP has an annual budget of $500 million, it has not been able to reach all children in India and now the second comprehensive multi-year plan (CMYP) for the duration of 2013–17 to achieve the program objectives of India’s universal immunization program seems to be lagging behind the targets [24,25]. Even though in India the immunization services are being provided free of cost in public health facilities, results show that 38 percent children are still either partially vaccinated or not vaccinated at all. Most importantly, though the coverage for most of the vaccines (BCG, measles and DPT) has shown an increase from the previous survey period, the coverage for polio vaccine witnessed a decline during the last decade. This declining trend of polio vaccine coverage (all the three doses of polio) is alarming. Though India achieved the polio free status in January, 2011 but to maintain the status is an ongoing challenge for India to prevent any outbreak of wild polio virus (WPV). To maintain the status, India has implemented the strategy to immunize every child through inactivated polio virus vaccine (IPV). To mention, true polio eradication means zero transmission of not only wild polio virus (WPVs) but also vaccine polioviruses [26]. The first phase of the eradication is the elimination of wild polio virus (WPVs) using oral polio vaccine (OPV) and elimination of vaccine polioviruses using inactivated poliovirus vaccine (IPV) is the second phase and India targeted the second phase [26]. And with an annual budget of $500 million through UIP, India is trying to reach every new born for full immunization.

The statistical analysis has made an important contribution in evaluating the recent estimates of the different components of child (12–23 months) immunization using advanced geo-statistical techniques. Such analysis gives a quick snapshot of disparities in immunization coverage at the sub-national levels which can help in developing priorities for those geographical units (districts) that need more attention. The analysis demonstrated spatial dependence, disparities and district level distal determinants of immunization coverage across India with special focus on the spatial dimension. This study identified the hotspots and cold spots of every component of child vaccination (BCG, DPT, Polio, Measles & full immunization) across the districts. Sharp regional disparities are evident in the coverage of different vaccination doses including full immunization with the presence of many cold spots in north-east and central part of India and hotspots in the southern parts. These results also highlighted the north-south inequality in the coverage of childhood vaccination. And the finding is consistent with the previous studies which attribute to the regional or state-level inequalities in the coverage of immunization due to the differential in socio-economic and health-care facility related factors like place of residence, wealth status, gender preference and health infrastructure [15,27,28].

The finding of this study suggested that women’s education had a positive impact on every vaccination (DPT, BCG, Polio, Measles) dose coverage, was found to be consistent with previous studies which assessed the link between maternal education and child health [13,15]. This study also revealed that breastfeeding children aged 6–23 months who also received adequate diet had a positive influence on the different components of child immunization. This can also be explained due to the fact that parents’ concern and awareness about child health usually increases the accessibility of available child health services for their children [12,13]. A positive association between health insurance schemes with different components of childhood immunization could be supported by the fact that provision of health insurance enables the women and the family to overcome the financial burden to avail the necessary health care facilities. This also indicates the preparedness of those households and awareness regarding the importance of holding a health insurance for an emergency health situation to seek health care. This could be also mentioned that purchasing health insurance does support the fact that those households are more of a well off families and concerned about their health care needs. And having health insurance enhances the opportunities for better child health care through access to free services and additional financial support to meet the indirect costs needed to access some of the health facilities [29]. Moreover, the coverage of households through insurance of household members provides indirect information about health care habits of the households.

Another key finding was that full ANC, PNC, institutional births, and mothers who received cash incentives under Janani Suraksha Yojana (JSY) scheme had a positive impact on full immunization and its components. This can be attributed to the fact that mothers participating in health care services usually become aware about the importance of immunization for her child’s health while discussing with the health professionals. Again, such schemes provide cash incentives to overcome various financial challenges in accessing health services. The schemes like JSY could also subsequently motivate mothers to join the immunization program by using health facility services. Previous studies have also established the linkage of maternal health care services and its influence on child health and immunization coverage in different country settings [14,30].

This study potentially contributes to the understanding of meso scale determinants of different doses vaccination coverage in districts of India. We used a recently published nationally representative factsheet data involving representative samples from all the 640 districts of India and thus, the results obtained from this analysis could be generalized across India. The study produced the snapshot of the most recent vaccination situation prevailing in India. At the same time, this study identified the meso scale risk factors of every vaccination doses of the ongoing full immunization programme for India which may help policy makers and planners to design new interventions to improve the coverage in the geographical coldspots where the coverage is eventually low compared to the other parts of India. Additionally, this study assessed the spatial heterogeneity of the different doses of vaccination using exploratory spatial data analysis (ESDA) methodology to estimate the risk factors considering the spatial distribution and dependence of immunization coverage of different doses. Furthermore, while giving away the estimates we used the National Family Health Survey (NFHS) which provides a nationally representative sample of women of childbearing age to collect the particular demographic information on fertility and mortality, anthropometry, family planning, maternity care, child feeding, vaccination, child morbidity and AIDS through a core questionnaire. And utilizing this data, estimates could be generated at national and at the sub-national levels. Thus taking the advantage of the sampling design of the data, district level estimation has been done to assess the determinants of low immunization coverage.

The study also has some limitations. Firstly, within a district level framework, this study examined the pattern of district level coverage of different vaccination doses and its meso scale determinants of low coverage across the districts. This could be further extended to lower disaggregated levels like the PSU or community levels to identify the intra-district disparity of vaccination among the children which may in turn help to find out the community level contextual factors determining the coverage of vaccination within districts. Secondly, though the study identified the geographical cold spots across India where the coverage of immunization is substantially low, but it did not investigate the phenomenon of the lower coverage of vaccination beyond the meso-scale framework. Third, the study is based upon cross-sectional data considering the children aged 12–23 months only across the districts and not all the children less than five years of age which would have helped to understand the extended scenario of vaccination among the children across India. Fourth, we used the health insurance variable and did not use the wealth variable which is the household level asset based information DHS provides to proxy the economic well-being of the household. To mention, the factsheet data lacked the district level aggregated information on proportion of poor or rich households which could be compiled from the unit level NFHS data. So, it could be exercised to look at the effect of economic condition (rich-poor distribution) prevailing across the districts and the related achievement in terms of vaccination coverage in those districts. As wealth based information are available from the unit level data of NFHS, controlling other covariates, the wealth effect could also be examined. Another basic limitation of NFHS data includes reporting and recall bias, particularly age and other retrospective data.

## Conclusion

Despite the fact that child vaccination coverage in India has seen a substantial increase over the last couple of decades, one-third of the children in India are not yet immunized. Results of our study clearly indicate regional disparities in childhood immunization levels. Though, the government and donor-funded programs have been consistently making efforts to improve the childhood immunization especially in the states of northern and central India, the focus on the north-eastern region has been limited. Even the existing program in the northern and central India does not seem to be very effective due to high number of cold spots in this region. This calls for more innovative models like incentivization and adoption of alternative ways of bringing mothers and children to the health facilities. The results indicating a positive association between childhood immunization and birth registration, institutional deliveries, having MCP card, and receipt of incentive under JSY schemes suggest that mothers’ exposure to health facilities can be one of the factors to positively influence the childhood vaccination upstream. This association is getting even stronger when the mother is educated reiterating the fact that increasing exposure of mothers to different MCH services via different health-care promotional activities help her in recognizing the need for availing health services not only for herself but for her children too. The Government may strengthen the MCH services via different health-care promotion activities. Although, JSY scheme shows a promising link between the components of child immunization, efforts are still needed to increase the coverage of this scheme as the coverage of this scheme is just 36 percent as per the recent data.
